# A scoping and web-based review of current practices and lessons learnt in development and sustainability of global health emergency medicine fellowships

**DOI:** 10.12688/mep.19503.1

**Published:** 2023-01-25

**Authors:** Haniya Khan, Alex McKnight, Kathleen Gamble, Lisa M Puchalski Ritchie

**Affiliations:** 1 Faculty of Medicine, University of Toronto, Toronto, Canada; 2 Department of Obstetrics and Gynecology, University of Toronto, Toronto, ON, Canada; 3 Global Health Emergency Medicine, Toronto General Hospital, Toronto, Ontario, Canada; 4 Li Ka Shing Knowledge Institute, St Michaels Hospital, Toronto, Ontario, Canada; 5 Department of Emergency Medicine, University Health Network, Canada, Toronto, Canada; 6 Department of Medicine, University of Toronto, Toronto, Ontario, Canada; 7 Institute of Health Policy, Management, and Evaluation, University of Toronto, Toronto, Canada

**Keywords:** Fellowship, global health, emergency medicine

## Abstract

**Background:** Despite significant interest in advanced global health training among Canadian emergency medicine trainees, only one global health emergency medicine (GHEM) fellowship existed in Canada at the time of this review. We conducted a scoping and web-based review to summarize the components of, and lessons learnt through development and implementation of global health emergency medicine fellowship programs to date, to inform program development.

**Methods:** We conducted a scoping and web-based review by systematically searching electronic databases from inception to 2021 for articles and websites (2022) describing global health emergency medicine training programs based in high income countries.

**Results:** From 2957 articles and 62 websites identified, eight articles and 43 websites were included in the review. Fellowships are generally structured as follows: 1–2 years duration curriculum including clinical skills, and course and field work focused on education, research or administration, funded by fellows’ clinical hours. Details on trainees’ experiences, international work, and program outcomes were lacking.

**Conclusions:** This review highlights the need for information on lessons learnt through development and implementation of GHEM fellowship programs, and experiences and outcomes of trainees to date, to inform program improvements to optimize the benefits of GHEM fellowship training.

**Registration:** Open science framework; https://doi.org/10.17605/OSF.IO/UAH35 February 19 ^th^, 2018.

## Introduction

Interest in global health training opportunities has grown rapidly over recent decades. In the United States, the percentage of medical students participating in global health experiences ranged from 15% to 27.1% from 2017 to 2021; a significant increase from 5.9% in 1978 ^1^. Over the same period, there has been an increase in the number of global health fellowships offered by North American academic institutions across a range of medical disciplines ^2, 3^. The first global health emergency medicine (GHEM) fellowship was introduced in 1994 ^4^. This number has increased steadily, with at least 39 currently available, accounting for over 40% of all global health fellowships offered ^5^.

Global health emergency medicine fellowships represent an avenue for academic institutions to deepen the learning experiences provided to trainees ^3, 6^. Such opportunities provide exposure to both a unique range of health conditions and spectrum of illness severity uncommon in high-income country settings ^3, 6^. In addition, trainees experience first-hand the challenges faced in providing clinical care within a resource limited healthcare setting. As a result, participants can gain clinical skills and may decrease their reliance on medical technologies while becoming more culturally sensitive and mindful of the resources available to them ^4^. However, fellowships vary significantly and little is known about best practices with respect to fellowship goals, structure, duration, supervision, and funding, in order to optimize trainees’ experience and program sustainability.

Despite significant interest in advanced global health training among Canadian medical trainees, only one emergency medicine-specific fellowship existed in Canada at the time of this review ^7^. To inform the development of a global health emergency medicine fellowship at the University of Toronto, we conducted a web-based and scoping review guided by the Johanna Briggs Institute guidance for scoping reviews ^8^. Our review addresses the following question: what are the current practices and lessons learnt to date in development and sustainability of global health emergency medicine fellowships for physicians during or post-residency based in high income countries?

## Methods

### Protocol

A protocol was developed for this review and registered with Open Science Framework ^9^. Reporting of findings was guided by the preferred reporting for systematic reviews and meta-analyses extension for scoping reviews (PRISMA ScR) ^10^.

### Eligibility criteria

We included websites that provided details of global health emergency medicine fellowships and articles that presented the experiences of GHEM fellowship developers, programs, faculty or trainees as narrative reports, or original research articles of any design, including formal review articles, qualitative and quantitative designs. Only reports of global health emergency medicine fellowships offered through academic institutions based in high-income countries were included. Countries were defined as high-income according to the World Bank’s World Country and Lending Group classification. Articles where reports of fellowships based in low- and middle-income countries that could not be separated out were not included.

### Information sources and literature search

A search strategy was developed in consultation with an experienced information specialist and peer-reviewed by a second information specialist using the Peer Review of Electronic Search Strategies checklist ^11^. The following databases were searched from inception to February 27, 2018 and subsequently updated to March 22, 2021, using medical subject heading (MeSH) headings and text words related to emergency medicine, fellowship, global health, and high income countries: OVID MEDLINE and EMBASE, CAB global health, CINAHL, HealthSTAR, ISIS Web of Science, EBM reviews, DARE abstracts, Scopus, psycINFO, ProQuest ERIC, LILACS, Google Scholar, Google, The British Library catalogue, ProQuest Dissertation, Open-Grey, New York academy of Medicine's grey literature, National Collaborative center for public health, OAIster, Health Canada, and Global Health Fellowships.org. Two databases planned for inclusion in our search were no longer accessible by our group at the time of the search, Sociological abstracts and NLM gateway. In addition, we searched reference lists of included articles (see *Extended data* for full MEDLINE search strategy ^12^).

Finally, we searched websites of global health and/or international health emergency medicine (EM) fellowship programs identified through the database search and a Google search (2022) with key words global and/or international health, emergency medicine, and fellowship.

### Article and website selection & data collection process

A screening checklist was developed and pilot tested by two authors (HK, AM) on 50 citations with three rounds necessary to reach >90% agreement. Articles were then screened independently by two authors (HK, AM) in two stages: titles and abstracts stage 1, and a full-text stage 2.

Discrepancies at each stage were resolved through discussion and involvement of the senior author as needed. A data abstraction form was developed and calibration exercise performed by two authors (HK, AM) to ensure consistency in data extraction. The calibration involved comparison and discussion of completed data abstraction forms for the first five articles. Data extraction was then done in duplicate by the same two authors, and discrepancies resolved through discussion and involvement of the senior author as needed. Information abstracted included population, concept and context characteristics and where applicable study characteristics and outcomes. Population characteristics of interest included: medical doctor (MD) stage and type of EM training. Concept characteristics of interest included: type of fellowship (formal/informal, accredited/not), when established, duration of the fellowship, curriculum, program structure, funding, location/type of GH/internal experience, supervision, number of trainees to date, lessons learnt in development/conduct of GH fellowships and program changes applied to the fellowships over time, fellows experiences, and perceived/known impact on fellows future careers. Context characteristics of interest included: fellowships based in high income country academic institutions. As no included articles reported studies appropriate for risk of bias assessment, risk of bias assessment was not done. Website screening was conducted in one stage, by two study team members independently (LPR and KG), and data extraction was conducted by KG and verified by LPR.

### Synthesis of results

Descriptive synthesis was conducted for all included articles and website data, and is reported narratively and in tabular form (see Table 1, and *Extended data table 2* ^12^).

**Table 1.** Characteristics of and outcomes reported in included articles.

Anderson, Philip D. 2005	International emergency medicine fellowships	Commentary Describes 9 available IEM Fellowship opportunities in the United States.	**Accreditation:** none of the fellowships described are accredited **Program Length:** fellowships are a minimum of 2 years in length. The longest continuously running fellowship described (at Loma Linda University) was established in 1994. **Curriculum:** fellowships described offer a combination of education, clinical practice, and hands- on experience needed to work in developing countries and health systems. Training may be tailored to the interests of individual fellows, but an integral component is working/interacting with international organizations and training in the development and implementation of emergency medicine services and public health interventions. **Funding:** most fellows' are paid by doing part-time clinical work in the ED at their home institution. **Fieldwork:** a broad range of international activities are described, ranging from response to humanitarian crises, to setting up treatment protocols for rural health workers, to developing EM training programs. **Lessons learned:** given that potential career paths are varied, IEM fellowship programs are most useful when they are flexible in accommodating individual backgrounds and needs. **Future prospects for graduates:** The majority of graduates are employed at academic institutions as academic emergency physicians. "Although no data have been published on the professional experiences of graduates of IEM fellowship programs, anecdotal experience suggests that a large percentage remains in academic EM practice, often with the goal of establishing new international programs based at United States academic medical centers."
Andescavage, S. M. 2011	Updated assessment of US-based international emergency medicine and global health fellowships	Study Abstract Reports finding of an electronic survey of EM program directors listed on the ACGME and American Osteopathic Association websites, with additional data collected via phone. The objective of the study was to determine the current status of IEM and GH fellowships available to EM residency graduates.	**Results:** Of 69 respondents to the survey, 18 EM programs confirmed current IEM or GH fellowships (26.1%). Of the 53 programs that did not have an IEM/GH fellowship, five responded ‘highly likely’ to have an IEM/GH fellowship within the next 3 years, and two responded ‘likely’ to have a fellowship within 3 years. **Curriculum:** 15/16 Fellowships offer an advanced degree (e.g., MPH, MS, MSPH). 10/16 fellowships have produced academic presentations and publications within 3 years. 9/16 fellowships offer observational rotations for foreign-trained medical doctors. **Fieldwork:** 40/53 programs without IEM/GH fellowships have at least one faculty member involved with international projects and offer residents the opportunity for international elective rotations. Fellows typically spend between 4 and 26 weeks in the field over the course of completing their Fellowship.
Bledsoe, Gregory H. 2005	Current status of International Emergency Medicine fellowships in the United States	Study Qualitative study using surveys and interviews to inform a summary of IEM fellowship opportunities offered to EM residency graduates in the United States. 127 accredited allopathic EM residency programs in the US were surveyed and 8 IEM fellowship programs were interviewed	**Results:** a total of 29 graduates of IEM fellowships were identified, of which 23 (79.3%) were in academic medicine and six (20%) were in community medicine. All remain active in international medicine. **Curriculum:** every IEM fellowship has implemented the proposed curriculum published by the SAEM consensus panel. Fellows participate in four areas outlined by the panel—clinical practice, education, research, and additional international projects. All IEM fellowships offer formal public health training and the possibility of obtaining an MPH degree; the 2-year programs require completing an MPH as part of the curriculum. **Fieldwork:** fellows participated in a variety of international projects including disaster response assessment, international pre-hospital education, and emergency services evaluation, as well as long-term training programs for the development of local Emergency Medicine providers, nutritional assessments, medical support for international government and non-government organizations, and post-conflict public health assessments. **Funding:** all fellowships were funded by a combination of clinical billing by the trainee and grants and scholarships specific for international work. The percentage of funding that came from grants and scholarships was program dependent with some programs more dependent upon clinical billing than others. **Number of graduates:** At the time of study, the number of graduates ranged from 0 to 7.
Crouse, Heather L. 2016	A Novel Approach to Combining Pediatric Emergency Medicine and Global Health Fellowships	Commentary Describes a 3–4 year PEM-GH Fellowship established in 2005 for general EM and pediatric residency–trained fellows.	**Curriculum:** the program involves a combination of formal and informal coursework, international fieldwork, and academic research. Fellows choose 1 of 3 curriculum pathways: 1) DTMH + public health certificate 2) DTMH + MPH or other approved advanced degree 3) MPH diploma or other approved advanced degree. All fellows attend the Health Emergencies in Large Populations Course. Formal coursework is supplemented with lectures and journal clubs that are available throughout the GH community at the hospital and medical school. There is a collaborative monthly seminar called Foundations in Global Health for professionals who are actively involved in GH work, and all fellows in the PEM-GH combined program are required to attend a quarterly Global Health Journal Club, created by the department of pediatrics for faculty, fellows, and residents conducting GH work. Fellows also have the opportunity to attend and deliver topic lectures on GH throughout the hospital and medical school. **Funding:** financial support comes from the department of pediatrics and the hospital. Funding for general IEM fellowship positions includes combination of institutional support, project-specific grants, and revenue from clinical billing. The department covers required travel and curricular components, including advanced degrees for the GH fellowship, as long as expenses met departmental cost constraints. To fund the fourth year of training, GH fellows are appointed as clinical instructors (with salary and benefits) for 12 months. During this time, their clinical hours are reduced by 25% compared to those of faculty appointed as full-time assistant professors to allow fellows time to complete requirements for the GH portion of their training. This funding scheme resulted from collaboration among administrators and academicians in an effort to deliver on the departmental mission within the institution’s margin. The program continues to grow because of philosophical and sustainable financial commitments and in-kind support from the department and hospital. **Fieldwork:** fellows spend 4 to 6 months abroad. Training opportunities in travel medicine, disaster response, refugee medicine, public health, human rights, PEM program development (including both curriculum and systems development), quality improvement, and EM provider training in both developed and developing countries are offered. Existing partnerships with collaborators in Africa and Latin America provide field opportunities for some fellows; other fellows may have established their own relationships prior to entering Fellowship training. **Supervision:** during time abroad, fellows receive on-site supervision from local experts and have regular contact with their U.S.-based faculty advisor(s). For their research, fellows have access to faculty mentors and advisors, research support, and training resources through the medical school and affiliated institutions. **Number of graduates:** since 2005 (to time of report), nine fellows (8 pediatric-trained and 1 EM trained) have completed or are enrolled in the PEM-GH fellowship, 3 have graduated. **Challenges:** creating a successful mission-driven yet financially sustainable GH fellowship in an era in which most academic institutions are experiencing reductions in grant funding, third-party reimbursement, and operating margins is the main challenge described.
Jacquet, Gabrielle A. 2014	Fellowships in international emergency medicine in the USA: a comparative survey of program directors' and fellows' perspectives on the curriculum	Study Survey administered to program directors, current fellows and recent graduates of the 34 US IEM fellowships. Response rate 60/78 (77%): 31/38 (82%) for PDs, 19/25 (76%) for current fellows, and 10/15 (67%) for recent fellowship graduates. Study aimed to examine program directors and fellows perceptions on whether IEM fellowships cover core curricular elements outlined earlier in the literature.	**Accreditation:** no fellowships are accredited. **Challenges:** the authors report that the majority of IEM fellows are not adequately exposed to core curriculum components proposed in prior studies. Deficiencies are the greatest in the fields of EMS and disaster medicine, and discrepancies between program directors and fellows are most evident in humanitarian aid, public health and disaster medicine. Authors note their findings beget the question whether IEM fellowship are trying to achieve too much within their time frame.
VanRooyen, M. J. 1997	International Health Fellowship: a proposed curriculum for emergency physicians	Commentary Describes the goals and objectives and development of an IEM fellowship at the University of Illinois.	**Program length:** the duration of the fellowship is 2 years. There is 1 fellow accepted per year, beginning in 1996. **Curriculum:** the fellowship is structured around the core curriculum for an MPH program, including epidemiology, biostatistics, health resources management, behavioral sciences, and community and environmental health. Elective courses in international health allow the fellow to study areas of specific interest. The faculty of the School of Public Health serves as mentors in independent study projects. The fellow will participate in additional course work in international health topics, including supplemental training in tropical medicine and disaster management, and will prepare grand round presentations and conduct lectures for teaching conferences in the College of Medicine and the School of Public Health. Research will emphasize innovations in international health. Research projects will be coordinated with faculty from the Department of Emergency Medicine and the School of Public Health. **Fieldwork:** the fellow will spend 3 (1- to 2-month blocks) months each year working abroad, spent in both developed and underdeveloped areas overseas and in both urban and rural settings. **Supervision:** field work will be arranged and coordinated by the Fellow under the supervision of the Fellowship Director. **Funding:** fellows are self-funded.
Collier, A. 2018	Global emergency medicine fellowship: Establishing a global health EM training program at Queen's University	Commentary Abstract Describes the establishment of a new global EM fellowship at Queens University in Canada.	**Accreditation:** the fellowship is not accredited. **Program length:** the 2-year program was established in 2017. **Curriculum:** the 2-year fellowship curriculum is divided between: 1) coursework to complete a Master of Public Health (MPH) Degree 2) fieldwork 3) relevant international emergency medicine training courses and 4) clinical work in the emergency departments at the Kingston Health Sciences Center. To date, the inaugural fellow has completed the Mission Craft Leadership in Disaster Relief course as well as a Humanitarian U Disaster and Response course, in addition to submitting a research grant as a co-principal investigator, starting coursework for an MPH degree and giving several invited lectures on humanitarian medicine. **Number of graduates:** the Queens Global EM fellowship admitted its first fellow in August 2017. **Fieldwork:** the fellow also travelled to Lebanon to support research in collaboration with aid organizations responding to the Syrian crisis. Upcoming fieldwork involves teaching at a newly established emergency medicine residency program in Haiti as well as a humanitarian crisis deployment.
Klesick, E. 2021	Global emergency medicine fellowships: Survey of curricula and pre-fellowship experiences	Study Survey of GH EM fellowships to describe GH EM fellowship curricula, experiences, and fellows career paths. 23 of 46 surveyed responded.	**Program structure:** 15 (68.2%) programs accepted one fellow per year, and seven (31.8%) accepted two fellows per year. Fellows averaged anywhere from 30–90 clinical hours per month, with an average of 61 hours (SD 13.5). **Program length:** one (4.6%) program was 1 year in length, while 21 (95.5%) programs were 2 years in length. **Fieldwork:** the amount of time fellows spent outside the US varied widely, from a minimum of 2–28 weeks (median 8 weeks, IQR ^6, 14^), to a maximum of 8–52 weeks (median 24 weeks, IQR (15,28). **Curriculum:** 19 (86.4%) programs offered a Master of Public Health (MPH) degree, while only three (13.6%) programs did not, with 14 (63.6%) programs requiring an MPH degree by time of graduation. 13 programs (59.1%) offered other degrees, such as a Diploma of Tropical Medicine and Hygiene, Master of Business Administration, Master of Science, and Global Health Certificate. Five (22.7%) programs reported that fellows participated in 0–1 research experiences during fellowship, six (27.3%) reported two research experiences, seven (31.8%) reported three research experiences, two (9.1%) reported four research experiences, one (4.6%) reported five experiences, and one (4.6%) reported over 10 research experiences during fellowship. 16 programs (73%) offered the Health Emergencies in Large Populations course, 11 (50%) a humanitarian response class, and one (5%) offered the American College of Emergency Physicians Emergency Medicine Basic Research Skills course. Seven programs dedicated time to point-of-care ultrasound training, ranging from 2–100 hours per year. A tropical medicine course was offered by 14 programs (64%) spanning 2–24 weeks (median 4 weeks, IQR 3.5-10.5 weeks). Other program offerings included attendance at the African Federation for Emergency Medicine and International Federation for Emergency Medicine meetings, disaster medical assistance team training and deployments, humanitarian emergency and hospital disaster simulation, physician leadership courses, and access to the World Health Organization. **Future prospects for graduates:** programs were asked to report how many recent graduates from their program were practicing in each of the following settings, 59% of graduates were reported to be working in US academic centers; 24% in US community practice settings, 9% for nonprofit agencies, and 9% in international clinical practice.

EM: Emergency Medicine; IEM: International Emergency Medicine; GH: Global Health; ACGME: Accreditation Council of Graduate Medical Education; SAEM: Society for Academic Emergency Medicine; DTM&H: Diploma in Tropical Medicine and Hygiene; MPH: Masters of Public Health; PEM: Pediatric emergency medicine.

## Results

### Literature search

**Figure 1.**
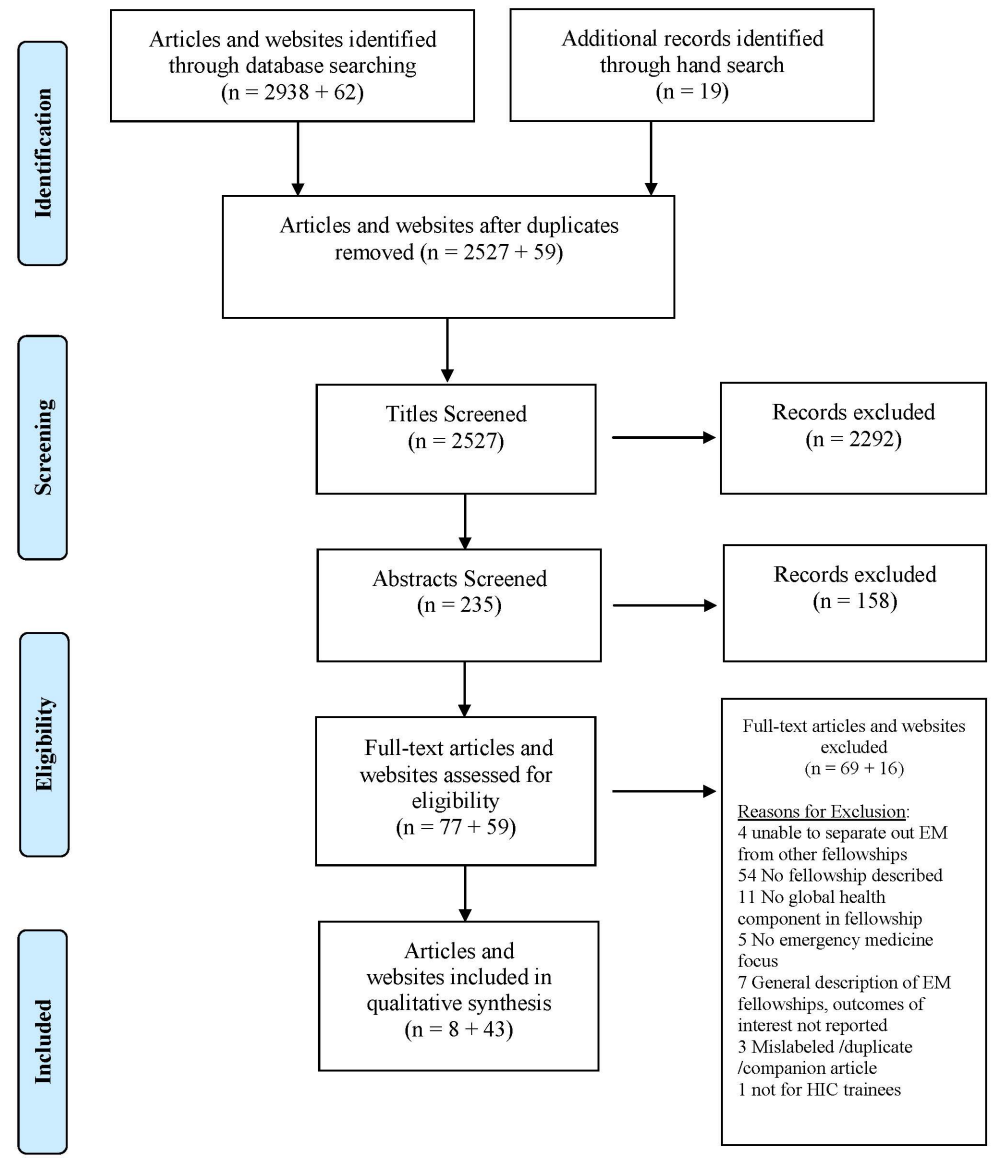
Prisma flow diagram.

A total of 2957 articles were identified through the database and hand search, and 62 websites were identified through the database and Google search. After duplicates were removed, 2527 titles and abstracts were screened, and 77 articles and 58 websites reviewed in full. A total of eight articles and 43 websites met eligibility criteria ( Figure 1).

### Characteristics of included articles and websites

Included articles were published between 1997 and 2021, and included six full text articles and two abstracts for which no full text article was available. Half of the articles presented results of survey studies and half were commentaries describing the need for and/or development of global health emergency medicine programs. The four studies conducted surveys, three with the objective of summarizing GHEM fellowship opportunities available in the United States ^2, 13, 14^ and one examining perceptions of program directors and fellows on whether GHEM fellowships cover core curricular elements outlined in the literature ^5^. All other included articles are commentaries describing the need for and/or the components of existing GHEM fellowships. Characteristics of one singular fellowship were described by three of the eight included articles; the remaining five articles summarize the state of GHEM fellowships in general or characteristics of multiple fellowships. All fellowships described or included in these eight articles originate in North America; one fellowship is based in Canada while all others are based in the United States. Similarly, with the exception of one program based in Canada, all included websites provided information on programs based in the United States.

### Review findings

***Program establishment, length, structure, funding, curriculum.*** Date of program establishment was provided by three articles and 16 websites. Program establishment dates ranged from 1994 to 2018, with only three established before 2000, seven established between 2000–10 and eight established between 2011–18 respectively.

Program length was reported in five of the included articles, with four ranging from 1–2 years ^6, 7, 13, 15^ and one, open to both pediatric and general EM-trained fellows, is 3–4 years long ^16^. All program websites reported program length which ranged from 12 to 26 months, with the majority of programs 2 years in length and the majority of (18 of 19) 1 year programs only available for fellows having previously obtained an appropriate advanced degree ^12^.

Half of the articles included describe the funding structure of the fellowship program(s). Most programs require fellows to work a certain number of clinical hours to fund their fellowship activities/salary ^2, 15, 16^. The description of the 3–4 year fellowship mentions financial support provided from the hospital department ^16^. One fellowship is described as self-funded by the fellow ^6^. Findings from the program website review were similar to the published articles, with 41 of 43 websites noting funding through clinical work ^12^. Provision of stipends or coverage of education costs such as tuition and travel, was noted in 16 websites ^12^.

Generally, programs align with the curriculum areas described by the Society for Academic Emergency Medicine (SAEM), which outlines three areas in which fellows should gain skills in at least one, in addition to clinical skills: administration, education, and research. Though the activities that fellows participate in under each vary, all fellowships discussed in the included articles mentioned fellows’ participating in all areas. The majority of articles (6/8) mention programs that include working towards a Master of Public Health degree as either a mandatory or optional route to fulfill the education component. This is sometimes supplemented with global health courses and/or journal clubs and other informal learning opportunities ^2, 6, 7, 13, 14, 16^. Similarly, all websites outline curriculum aligning with SAEM recommendations ^12^. The vast majority of programs require completion of a masters degree (37/43) (most commonly a master of public health) as part of 2 year programs unless an appropriate degree has been previously completed, with completion of masters degree an option in the remaining 2 year programs ^12^.

***Global health/international experience and fellow supervision.*** All fellowships described in these articles include an international fieldwork component, though the duration and nature of the fieldwork varies. The shortest duration reported is 2 weeks while the longest is 52 weeks ^13^. Fieldwork opportunities are mostly described as flexible, with opportunities to attach to ongoing international projects that the program is engaged in, or develop their own fieldwork experience based on the fellow’s existing relationships. The majority of programs (32/43) require an international fieldwork component, with eight programs noting international experience options but without clarity in whether this is a requirement ^12^. As noted in the published articles, fieldwork opportunities described on program websites vary widely in duration (2 to 12 months), and focus (clinical, research, education), although the majority include clinical work as part of the international experience ^12^. Established international partnerships were noted by 22 programs, with several noting that other international sites may be considered ^12^.

Only two articles discuss supervision of fellows ^6, 16^. Both describe faculty mentorship at the fellow’s home institution. In addition, Crouse mentions that fellows are paired with a local supervisor during their fieldwork while maintaining regular contact with their faculty mentor at their home institution ^16^. As with the published articles, supervision is infrequently discussed in program websites, with eight noting supervision/mentorship to be provided by local faculty with appropriate expertise, and none clearly outlining on-site supervision during international fieldwork ^12^.

***Number of trainees to date.*** The total number of GHEM fellowship trainees to date was noted by two included articles and ranged from 0–7 at the time of publication ^2, 16^. Similar to the published articles, few programs reported information on the number of trainees to date, with numbers ranging from 2–31 for the three programs reporting totals ^12^.

***Fellows’ experiences in the program, perceived impacte on fellows careers.*** The articles did not discuss graduates’ career trajectories in-depth, however two studies ^2, 13^ surveyed participants to find out where graduates were working following their fellowships. Bledsoe found that out of a total of 29 graduates of international emergency medicine (IEM) fellowships identified, all remain active in international medicine, however it is unclear whether the author interviewed the graduates to obtain this information, or received this information from programs ^2^. Klesick received information from the fellowship programs, stating that 59% of graduates were working in US academic centers; 24% were working in US community practice settings, 9% were working for nonprofit agencies, and 9% were working in international clinical practice ^13^. Only one website provides information on graduates career trajectories, noting that three graduates work in academic pediatric emergency medicine global health programs and are engaged in international work ^12^. Quotes from graduates regarding specific activities undertaken and lessons learnt during their fellowship training were included in two websites. While quotes were quite positive there are too few to draw meaningful conclusions about fellows’ experiences.

***Lessons learned in development/conduct of GH fellowships overtime; program changes over time.*** Half of the included articles (4/8) identified several salient lessons from their experience either directly participating in an existing GHEM fellowship or evaluating the landscape of GHEM fellowships. All noted that the available GHEM fellowships at the time of their writing were considerably varied; the global health emergency medicine field is vast, and fellows are presented with many different foci within the field (e.g., disaster medicine, humanitarian relief, developing emergency systems, public health, and others). The authors note that this variability in focus can result in fellows that are not adequately prepared in any area. “Neither program directors (PDs) nor fellows believe that fellows have achieved an adequate level of capacity (minimum score of 3) in any of the six core curriculum components studied: EM systems development, EM education development, humanitarian aid, public health, EMS and disaster medicine. After completing a 1- or 2-year IEM fellowship, it is unlikely that a graduate would master all six core curriculum areas. However, the fact that having an average score of 3 (perfect amount) in any of the six curriculum areas was reported neither by PDs or fellows is an interesting finding which begets the question whether IEM fellowship are trying to achieve ‘too much’, stacking the deck with more core competencies than they can achieve within the allocated time frame” ^5^.

Crouse notes that a significant challenge can lie in establishing a sustainable funding structure, in an era of reduced grant funding and smaller operating margins for academic institutions ^16^. Ensuring that a fellowship program is supported from within the institutions, philosophically and financially, is vital to ensuring the long-term health of the fellowship.

None of the include articles reported changes to the program overtime. Similarly, none of the websites discussed lessons learned or changes to programs overtime.

## Discussion

The goal of this scoping review was to summarize components of existing global health emergency medicine fellowship programs and lessons learnt by developers, faculty and trainees in these programs. Our findings revealed some information on the structural components of the fellowship programs such as duration, supervision, funding structure, and broad curricular elements. Notably, though, our review revealed that the experience of developers, faculty and trainees of existing global health emergency medicine fellowship programs have not been rigorously reported in the literature. Data on the experiences of trainees, details of trainees’ international exposure, and program outcomes is lacking in the literature. This poses a challenge for institutions attempting to develop global health emergency medicine fellowship programs as the literature excludes experiential lessons that could be applied to improve new and existing programs. 

### Fellowship structure

There is discussion in the literature around the appropriate design of a global health emergency medicine fellowship. Specifically, the discourse centered on whether fellowships should have standardized vs. flexible curricula, and exposure-based (exposure to all pre-defined curricular components) vs. proficiency-based (focus on select curricular components) curricula ^5^. Additionally, the literature revealed that many trainees are not actually aware of fellowship curricula prior to starting a program ^5^. Given the diverse motivations of trainees completing global health emergency medicine fellowships, a one size fits all approach may not be appropriate. Anderson *et al.* described that “fellowship programs are most useful when they are flexible in accommodating individual backgrounds and needs” ^15^. As such, fellowship programs may benefit from developing specific curricular goals in collaboration with individual trainees. Although this process may prove to be more resource intensive, it may lead to improved outcomes for trainees and programs.

### Trainee experiences

The literature is lacking an account of trainee experiences in global health emergency medicine fellowship programs. Only three of the eight articles included and one of the websites in this review reported fellowship graduates’ career trajectories. This is a critical piece of data that should be better documented to assess the impact of fellowship training programs. Additionally, it would be important to learn from trainees whether the skills, exposure, and knowledge they gained from their fellowships proved beneficial in their future roles and whether there are aspects of training provided by existing programs that could be improved.

### Fellowship challenges

The most frequently described challenges in initiating and maintaining global health emergency medicine fellowships outlined in the literature were: (a) lack of stable funding, (b) scarcity of international partnerships, (c) challenges with fellow supervision on international experiences, and (d) low levels of institutional support. All articles and websites included in this review that provided information on funding models described that trainees were financially supported by revenue from their clinical work, project-specific grants, and in rare cases institutional funds. An understanding of programs and fellows’ experiences would provide greater appreciation for the benefits and drawbacks of the various funding models. While 22 websites note ongoing international relationships which provide fieldwork opportunities to trainees, relatively little detail is provided about the fieldwork experiences of trainees to date, such as knowledge and skills acquired. Additionally, although adequate support and supervision are essential to optimizing fieldwork experiences, data on supervision is largely omitted from the data sources reviewed. Institutions offering global health emergency medicine fellowships should initiate, foster, and sustain partnerships with organizations in low- and middle-income countries to allow for an organized and sustainable exchange of knowledge and resources. Given trainees’ increasing interest in global health emergency medicine, it is surprising to see a lack of institutional support described as one of the challenges in initiating and maintaining such fellowships. Future work should define the types of institutional support required to succeed.

### Strengths

This scoping review employed a rigorous and systematic methodology, was prospectively registered, and used predetermined inclusion and exclusion criteria. Additionally, the review employed a broad search strategy, which included a search of grey literature sources and program websites, and a search update to identify all relevant studies.

### Limitations

The main limitation of this review is the lack of reporting of evaluation of existing programs and sharing of lessons learnt to date. As a result, data for some areas of interest were not available or lacked the detail necessary to draw meaningful conclusions. Such detail may have been gained through direct contact with programs, to interview program staff and review departmental reports related to concepts of interest. Future work to address these gaps through mixed methods research with program developers, faculty and trainees, to understand experiences and impacts on fellows’ careers to inform program improvements to optimize program benefits to fellows and support continued growth of GHEM as a subspecialty, is needed.

## Conclusions

Despite significant interest in advanced global health training among emergency medicine trainees and practitioners and the growth in availability of GHEM fellowships in recent decades, this review highlights that information to guide development and implementation of such programs is limited. Future work to learn from the experiences of program developers, faculty and trainees to date, including gaps in training and impacts of current training on future career trajectories is needed to inform program improvements to optimize the benefits of GHEM fellowship training to trainees and to advance the GHEM as a subspecialty.

## Data Availability

All data underlying the results are available as part of the article and no additional source data are required. Open Science Framework: Extended Data file. https://doi.org/10.17605/OSF.IO/N3MZV ^12^. This project contains the following extended data: Extended Data revised prisma checklist, full medline search strategy, results table 2.docx (Full MEDLINE search strategy, Results table 2: websites included and outcomes reported) Open Science Framework: PRISMA-Scr checklist for ‘A scoping and web-based review of current practices and lessons learnt in development and sustainability of global health emergency medicine fellowships’, Extended Data File. https://doi.org/10.17605/OSF.IO/N3MZV ^12^. Data are available under the terms of the Creative Commons Zero "No rights reserved" data waiver (CC0 1.0 Public domain dedication).
