# Supplementary material for: A scoping and web-based review of current practices and lessons learnt in development and sustainability of global health emergency medicine fellowships
Source: MedEdPublish (2016). 2023 Jul 17;13:6. Originally published 2023 Jan 25. [Version 2] doi: 10.12688/mep.19503.2 (PMC12784044; doi:10.12688/mep.19503.2)
Supplement: Peer review history

# Reviewer report: Reviewer response for version 2

**DOI:** 10.21956/mep.21133.r44218

**Published:** 2026-01-08

**Authors:** Ashti Doobay-Persaud, Arthi Kozhumam

**Affiliations:** 1 Northwestern University Feinberg School of Medicine Galter Health Sciences Library, Chicago, Illinois, USA

Thank you to the authors for pursuing an important topic in global health education for the clinical health professional especially in graduate medical education in the emergency medicine field. The scoping review methods are well-described, with reporting of the protocol on OSF and adherence to PRISMA guidelines, the comprehensive search strategy, and described data extraction procedures. One limitation of reproducibility is the lack of full search criteria or a search string that could be considered for addition in an Appendix. The authors conducted and present largely a descriptive narrative analysis, with qualitative findings thoroughly described and supported by counts and ranged as applicable. Interpretation of findings is framed appropriately within evidence limitations, including the predominance of US EM fellowships. The authors' conclusions are well-supported by the results, primarily stating a current focus on program structure and funding with limited focus on other aspects. Limitations are appropriately acknowledged. Below are some minor remaining comments for consideration:

 Introduction:

1. While the introduction establishes the context for the study, it could be strengthened by designating the unique contribution of a scoping review relative to prior survey/commentary literature in the Canadian context. A more explicit explanation of how/why a scoping and web-based review adds methodological value and rigor beyond existing work would improve clarity.
2. Additionally, the inclusion of findings from primarily US-based programs is highlighted mostly in the limitations; given the focus on the Canadian context but the predominance of included findings from the US, the intro could better justify why findings from these other programs could/will meaningfully inform Canadian fellowship development.
3. Consider briefly stating why a scoping review (rather than a systematic review for example) is the most appropriate methodological approach for the stated research question.

Methods:

1. Inclusion of the search strategy in-text or as an Appendix would improve transparency.
2. While the database search is well-described, the web-based review component could be clarified: how were data standardized, how was missing/unclear information handled, and when were sites searched/were they real-time or time-stamped or archived?

Results:

1. While numerous program features are catalogued (e.g., length, funding models, curriculum alignment), the narrative at times reads as like an inventory list; would recommend further emphasis on which characteristics appear most salient for each topic area like sustainability or trainee experience.
2. With significant differences in reliability and depth between peer-reviewed and web-based resources, explicit labelling of findings from each source type could help readers better assess the strength of the evidence provided. 

Discussion/Conclusion:

1. The conclusions are appropriately cautious and aligned with the descriptive findings; however, the discussion could be strengthened by more explicitly linking specific findings to actionable recommendations. For example, the authors could more directly discuss implications for equity, trainee workload, or long-term program sustainability.
2. The discussion would additionally benefit from more concrete suggestions for standardized reporting or evaluation frameworks that future fellowship programs could adopt.

Are the rationale for, and objectives of, the Systematic Review clearly stated?

Yes

Is the statistical analysis and its interpretation appropriate?

I cannot comment. A qualified statistician is required.

Are sufficient details of the methods and analysis provided to allow replication by others?

Partly

Are the conclusions drawn adequately supported by the results presented in the review?

Yes

Reviewer Expertise:

Global health and education, not a statistical expert

We confirm that we have read this submission and believe that we have an appropriate level of expertise to confirm that it is of an acceptable scientific standard, however we have significant reservations, as outlined above.

# Reviewer report: Reviewer response for version 2

**DOI:** 10.21956/mep.21133.r34163

**Published:** 2023-08-08

**Authors:** Melissa L Langhan

**Affiliations:** 1 Section of Pediatric Emergency Medicine, Departments of Pediatrics and Emergency Medicine, Yale University School of Medicine, New Haven, USA

Thank you for sharing these revisions. I approve this article for indexing

Are the rationale for, and objectives of, the Systematic Review clearly stated?

Partly

Is the statistical analysis and its interpretation appropriate?

Not applicable

Are sufficient details of the methods and analysis provided to allow replication by others?

Yes

Are the conclusions drawn adequately supported by the results presented in the review?

Yes

Reviewer Expertise:

Pediatric emergency medicine fellowship

I confirm that I have read this submission and believe that I have an appropriate level of expertise to confirm that it is of an acceptable scientific standard.

# Reviewer report: Reviewer response for version 2

**DOI:** 10.21956/mep.21133.r34164

**Published:** 2023-07-27

**Authors:** Stacey M. Kassutto

**Affiliations:** 1 Department of Medicine, Division of Pulmonary, Allergy and Critical Care, Perelman School of Medicine, University of Pennsylvania, Philadelphia, PA, USA

This remains a study based upon a small number (8) of published papers and a review of programmatic websites to describe the current state of EM GH fellowships. I agree with the other reviewer that the few number of papers published that are included in this review remains a significant limitation of this study. The authors have addressed the concerns that can be addressed but fundamentally the conclusions that can be drawn from the studies included are quite limited. There remain a few minor typos throughout the manuscript.

Are the rationale for, and objectives of, the Systematic Review clearly stated?

Yes

Is the statistical analysis and its interpretation appropriate?

Not applicable

Are sufficient details of the methods and analysis provided to allow replication by others?

Partly

Are the conclusions drawn adequately supported by the results presented in the review?

Yes

Reviewer Expertise:

areas of research include curriculum development, simulation, technology, virtual reality

I confirm that I have read this submission and believe that I have an appropriate level of expertise to confirm that it is of an acceptable scientific standard, however I have significant reservations, as outlined above.

# Reviewer report: Reviewer response for version 1

**DOI:** 10.21956/mep.20896.r33572

**Published:** 2023-06-09

**Authors:** Melissa L Langhan

**Affiliations:** 1 Section of Pediatric Emergency Medicine, Departments of Pediatrics and Emergency Medicine, Yale University School of Medicine, New Haven, USA

The authors conducted a scoping review of websites and the literature regarding EM GH fellowship programs with the goal of summarizing components of these programs and lessons learned. 

 The authors state in their introduction that there is "significant interest" in advanced GH training without providing any evidence for this statement. Do we know how many residents are interested and/or applying for GH fellowships? Are there too many and not enough programs? It would be helpful for the authors to include data on the actual interest and need.

 While I understand that the authors are looking to establish an EM based GH fellowship, it seems like they may have gained additional information by a more comprehensive review including non-EM based GH fellowships as well, since the majority of fellowship are outside of EM programs. 

 As supported by the finding of only 8 articles on this topic, 4 commentaries and several summaries of existing programs (many about the same program), it is unusual for program directors to attempt to publish descriptions of their programs, nor for most medical journals to have an interest in publishing such data given that it is not adding to the general knowledge base of medicine. While established programs may publish on innovations, this is less likely to be found among a younger specialty such as EM GH. This is an important limitation to this study. The authors attempted to supplement this data by searching program websites, however the level of detail provided on program components is insufficient for readers to use in program development, the main goal of this study. Furthermore, the impact of both the national and institutional environments on program development has not been well established. Since nearly all programs are in the US, this information is not necessarily generalizable to other countries.

 What this review has highlighted is that given a lack of accreditation to these programs, there are no governing bodies to assess programmatic outcomes. Thus we do not have sufficient data on any of the current programs to indicate that they are actually successful in their goals. This makes the publication of data difficult. A perspective piece on the lack of data and outcomes of these programs would be a timely and relevant addition to the literature.

 One specific note, the authors should revise their description of the included PEM-GH combined fellowship. While the authors state that this is a 3-4 year fellowship, this is because US PEM training is required to be 2-3 years depending on your primary residency training. Thus the GH portion of this program (which is likely not accredited) is more likely 1 additional year.

Are the rationale for, and objectives of, the Systematic Review clearly stated?

Partly

Is the statistical analysis and its interpretation appropriate?

Not applicable

Are sufficient details of the methods and analysis provided to allow replication by others?

Yes

Are the conclusions drawn adequately supported by the results presented in the review?

Yes

Reviewer Expertise:

Pediatric emergency medicine fellowship

I confirm that I have read this submission and believe that I have an appropriate level of expertise to state that I do not consider it to be of an acceptable scientific standard, for reasons outlined above.

# Response

**Published:** 2023-07-08

**Authors:** Lisa M Puchalski Ritchie

**Affiliations:** Li Ka Shing Knowledge Institute, St. Michaels Hospital, Toronto, Ontario, Canada

We thank the reviewer for their careful review and comments on this paper.  We were unable to find Canadian data in the general medical literature, however, we have included data from a GH training program for residents and fellows based at the University of Toronto, that has shown sustained and increasing interest in this advanced GH training opportunity. While it is possible that further information may have been gained through inclusion of data on non-EM global health fellowships, as EM in many international settings in is early stages of development or not recognized as a specialty, we felt that this may create unique challengs and opportunities for GHEM fellows particularly with respect to their international experiences, and therefore chose to limit our review to EM specific programs. We have noted this is the limitation section. Given published calls for research to evaluate GH trainee expereinces and outcomes for over a decade, we feel that our finding of a continued lack of published/accessible data is of value in further highlighting the need for such data to both improve existing programs and to support development of new programs. We added a future directions section to further highlight this point and to outine areas for future research to begin to address research gaps identified by our review. While many of the lssons from US sites will likely be of value to other high income country programs, we agree that some may be of less value to non-US sites and have noted this as a limitation in the revised version. We agree that program evaluation data is lacking, at least in the published/accessible literature. Given the recommendations for research to evaluate trainee experiences and outcomes ina variety of aspects of GH training, published in the 2013 emergency medicine consensus proceedings, we feel that the lack of published data found in our review, is of value in highlighting the ongoing need for such evaluation reasearch. And as noted above we have added a future directions section to further highlight this point and outline areas for future research to begin to address the research gap identified by our review. We have clarified that the PEM-GH program is composed of 2-3 years of general PEM training plus an additional year for the GH component, with the GH training spread throughout the full 3-4 year progam.   As Noted

# Reviewer report: Reviewer response for version 1

**DOI:** 10.21956/mep.20896.r32922

**Published:** 2023-02-15

**Authors:** Stacey M. Kassutto

**Affiliations:** 1 Department of Medicine, Division of Pulmonary, Allergy and Critical Care, Perelman School of Medicine, University of Pennsylvania, Philadelphia, PA, USA

The authors describe a scoping and web-based review of global health emergency medicine fellowships in the US and Canada. Their results are based on 8 articles and 43 websites used to describe fellowship characteristics including length, funding mechanisms, curricular content, international experience and supervision.

 Overall the article is well written (there are a few typos that will need to be addressed). The manuscript establishes that the literature on this topic is quite limited, given that only 8 articles were ultimately included in the study and that more work is needed to better delineate the current state and needs for this curricular area of interest. It would be helpful if the authors better delineated the exclusion criteria that led to the exclusion of 2292 records. Figure 1 outlines exclusion criteria for the 69 articles excluded later in the process but there is no description of the exclusion criteria from initial screening. The methods mention that a screening checklist was developed and pilot tested but the checklist itself is not further described.

Are the rationale for, and objectives of, the Systematic Review clearly stated?

Yes

Is the statistical analysis and its interpretation appropriate?

Not applicable

Are sufficient details of the methods and analysis provided to allow replication by others?

Partly

Are the conclusions drawn adequately supported by the results presented in the review?

Yes

Reviewer Expertise:

areas of research include curriculum development, simulation, technology, virtual reality

I confirm that I have read this submission and believe that I have an appropriate level of expertise to confirm that it is of an acceptable scientific standard, however I have significant reservations, as outlined above.

# Response

**Published:** 2023-07-08

**Authors:** Lisa M Puchalski Ritchie

**Affiliations:** Li Ka Shing Knowledge Institute, St. Michaels Hospital, Toronto, Ontario, Canada

We thank the reviewer for their careful review and comments on this article. We have addressed the typos in the revised version. In addition, we have revised the description of the exclusion criteria and have added a description of the format and content of the screening checklist.
